# Expression Analysis of *SOCS* Genes in Migraine

**DOI:** 10.3389/fnmol.2021.725048

**Published:** 2021-09-27

**Authors:** Soudeh Ghafouri-Fard, Kasra Honarmand Tamizkar, Arezou Sayad, Mohammad Taheri, Mohammad Samadian

**Affiliations:** ^1^Department of Medical Genetics, School of Medicine, Shahid Beheshti University of Medical Sciences, Tehran, Iran; ^2^Men’s Health and Reproductive Health Research Center, Shahid Beheshti University of Medical Sciences, Tehran, Iran; ^3^Skull Base Research Center, Loghman Hakim Hospital, Shahid Beheshti University of Medical Sciences, Tehran, Iran

**Keywords:** SOCS, expression, migraine, aura, without aura

## Abstract

Migraine is a complex neurological condition affecting a large proportion of persons. Dysregulation of several immune-related transcripts has been noted in migraineurs suggesting an immune-based background for this condition. We measured expression levels *suppressor of cytokine signaling* (*SOCS*) genes in the venous blood of migraineurs compared with controls. *SOCS1* was down-regulated in patients without aura compared with controls [Ratio of mean expression (RME) = 0.08, *P* value < 0.001]. This pattern was also detected among female subgroups (RME = 0.06, *P* value = 0.010), but not among male subgroups (RME = 0.22, *P* value = 0.114). Expression of *SOCS1* was significantly higher in patients with aura compared with those without aura (RME = 5.89, *P* value = 0.037). Meanwhile, expression of *SOCS2* was lower in migraineurs with aura compared with controls (RME = 0.03, *P* value < 0.001). In addition, this gene was under-expressed in patients without aura compared with controls and in both sex-based subgroups of this group of patients (RME = 0.01, *P* value < 0.001 for all comparisons). However, its expression was higher in male patients with aura compared with those without aura (*P* value < 0.001). For *SOCS3*, we detected a lower level of expression in patients without aura compared with controls (RME = 0.07, *P* value < 0.001). However, the expression of *SOCS3* was higher in patients with aura compared with those without aura (RME = 7.46, *P* value = 0.001). *SOCS5* was down-regulated in patients without aura compared with controls (RME = 0.10, *P* value < 0.001). Expression of this gene was also lower in patients with aura compared with controls (RME = 0.03, *P* value < 0.001), and in male patients of this group compared with controls (RME = 0.03, *P* value = 0.004). On the other hand, expression of *SOCS5* was higher in male patients with aura compared with sex-matched patients without aura (RME = 6.67, *P* value = 0.001). *SOCS2* levels could appropriately differentiate migraineurs from healthy subjects. The current study suggests the role of *SOCS* genes in the pathoetiology of migraine.

## Introduction

The suppressor of cytokine signaling (SOCS) constitutes a family of proteins that potently inhibit the cytokine-activated Janus kinase (JAK)/signal transducer and activator of transcription (STAT) cascade (Cooney, 2002). The negative feedback regulated by SOCS proteins precludes the disproportionate production of cytokines, therefore protects the host from their harmful effects (Alston and Dix, 2019). This family includes SOCS1–7 proteins as well as cytokine-induced STAT inhibitor (CIS) that principally regulate the response of inflammatory cells to cytokines (Huang et al., 2020). In addition to their role in the regulation of JAK/STAT, SOCS proteins have been found to regulate Toll-like receptor (TLR) signaling (Kinjyo et al., 2002; Posselt et al., 2011). Moreover, certain members of this family have been shown to affect the activity of other signaling pathways. Examples are regulation of epidermal growth factor receptor (EGFR) signaling by SOCS5 (Kario et al., 2005), ubiquitination of a number of receptors for cytokines, growth factors, and hormones by SOCS3, and the ability of this protein in binding with indoleamine dioxygenase (IDO; Orabona et al., 2005, 2008). Additionally, SOCS3 has an established role in the regulation of sensitivity to insulin through inducing proteasome degradation of its receptor (Hilton et al., 2000). Based on these diverse roles, SOCS proteins are involved in the pathoetiology of a wide range of human disorders including malignant conditions, autoimmune disorders, and viral infections (Inagaki-Ohara et al., 2013; Liang et al., 2014; Huang et al., 2020).

Migraine is a complex disorder with unclarified pathogenic mechanisms. Among several hypotheses suggested to explain its pathogenesis, the hypothesis of immune system alteration has gained attention (Bruno et al., 2007). Cytokines as chief regulators of the immune pathways have been found to be broadly expressed in the central nervous system particularly by neurons, demonstrating their impact on neuronal receptors (Bruno et al., 2007). Moreover, cytokines have been recognized to function as pain mediators and participate in neurovascular inflammation (Hung et al., 2017). Chemokines have also been shown to activate trigeminal nerves, thus contributing in the migraine pain (Bruno et al., 2007). Based on the above-mentioned evidence, we hypothesized that dysregulation of SOCS proteins might contribute to the pathoetiology of migraine. Therefore, we measured expression levels of *SOCS* transcripts in the peripheral blood of migraineurs compared with healthy persons.

## Materials and Methods

### Enrollment of Cases and Controls

Venous blood samples have been collected from 116 migraineurs (63 migraineurs with aura and 53 migraineurs without aura; age [mean ± standard deviation (SD)] = 37.47 ± 11.94) and 40 control subjects. Migraineurs were referred to the Neurology Clinic of Imam Hossein Hospital, Tehran, Iran, during 2019–2020. None of them had a headache at the time of blood sampling. Migraineurs were assessed using the diagnostic criteria of the International Headache Society (third edition; Olesen, 2018). Control subjects had no history of migraine headaches. The presence of any systemic disorder such as malignancy, diabetes mellitus or an autoimmune disease was considered as an exclusion criterion. Moreover, a history of serious head injury or any ischemic attacks were considered as exclusion criteria for the enlistment of cases and controls. The study protocol was verified by the ethical committee of Shahid Beheshti University of Medical Sciences. All case and control subjects signed informed consent forms.

### Expression Assay

PicoPure™ RNA Isolation Kit (Thermo Fisher Scientific) was used for extraction of total RNA from venous blood samples. Afterward, cDNA was made from RNA by using the cDNA synthesis kit (Smobio, Taiwan). Relative expressions of *SOCS* genes were quantified in all obtained specimens using the qRT-PCR kit (GeneDireX, Miaoli County, Taiwan). PCR was accomplished in LightCycler^®^ 96 instrument in duplicate. Primer sequences and PCR conditions were similar to our previous study (Ghafouri-Fard et al., 2018).

### Statistical Methods

Statistical analysis was accomplished using the R programming language. Expressions of *SOCS* genes were calculated using Ct and efficiency parameters using the following equation: $\frac{amp_{Target\;gene}^{-CT_{Target\;gene}}}{amp_{Normalizer\;gene}^{-CT_{Normalizer\;gene}}}$ where amp indicates the efficiency of amplification. Log-transformed values of expression data were used for further analysis. The mean of these values were compared between groups. In order to have an estimate of expression values among cases and controls, we also calculated the ratio of mean expression values (RME) using the following formula: Mean value of gene expression in cases/Mean value of gene expression in controls. Three comparisons were performed between groups (Aura vs. control, Without Aura vs. control, Aura vs. Without Aura). The normality of data was checked using the Shapiro–Wilk test which showed that expressions data do not follow a normal distribution. The Kruskal–Wallis test as a nonparametric test was used to see if the medians of these three groups are significantly different for each of the genes. A *post hoc* analysis (Dunn’s test) was performed for those with significant *P* values. *P*-values of two-by-two comparisons were adjusted using the Benjamin–Hochberg method. This workflow of analysis is done for each gender as well. The Spearman correlation coefficient was measured to evaluate correlations between expressions of *SOCS* genes. Receiver operating characteristic (ROC) curve was depicted for each gene and area under curve (AUC) value was measured.

## Results

**Figure 1:**
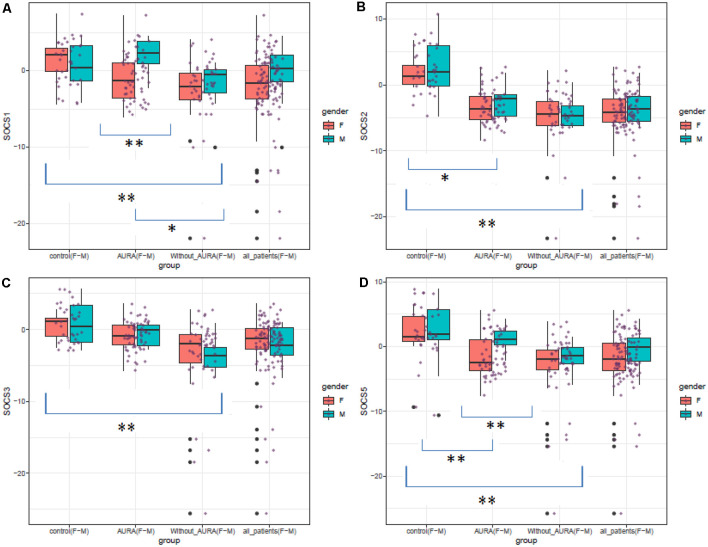
Expression levels of *SOCS* genes in patients (total patients, patients with aura, and patients without aura) and controls based on their gender (Median and interquartile range are shown. Purple points show each expression level. Outliers are also shown by black points. *P value < 0.05, **P value < 0.001).

Figure 1 shows expression levels of *SOCS* genes in patients (total patients, patients with aura, and patients without aura) and controls based on their gender.

The Shapiro–Wilk test rejected the hypothesis that expressions follow a normal distribution. Therefore, Kruskal–Wallis which is a nonparametric test was used to see if medians of these three groups are significantly different for each of the genes. For all *SOCS* genes, Kruskal–Wallis showed a significant change amongst groups, therefore, a *post hoc* analysis (Dunn’s test) was performed and *P* values of two-by-two comparisons were adjusted using the Benjamin–Hochberg method. This workflow of analysis is done for each gender as well. The results of the Kruksal–Wallis test for all subjects are shown in Table 1.

**Table 1:** Relative expressions of SOCS genes in patients compared with controls (RME: ratio of mean expression).

	All subjects		Females		Males	
Genes	Kruskal–Wallis chi-squared	*P* Value	Kruskal–Wallis chi-squared	*P* Value	Kruskal–Wallis chi-squared	*P* Value
*SOCS1*	16.635	0.000	8.997	0.011	5.618	0.060
*SOCS2*	61.627	0.000	19.080	0.000	24.501	0.000
*SOCS3*	24.634	0.000	11.556	0.003	12.344	0.002
*SOCS5*	33.444	0.000	10.821	0.004	12.628	0.001

*SOCS1* was down-regulated in patients without aura compared with controls (Ratio of mean expression (RME) = 0.08, *P* value < 0.001). This pattern was also detected among female subgroups (RME = 0.06, *P* value = 0.010), but not among male subgroups (RME = 0.22, *P* value = 0.113). Expression of *SOCS1* was significantly higher in patients with aura compared with those without aura (RME = 5.89, *P* value = 0.037).

*SOCS2* was down-regulated in migraineurs with aura compared with controls (RME = 0.03, *P* value < 0.001), and in both sex-based subgroups. In addition, this gene was down-regulated in patients without aura compared with controls and in both sex-based subgroups of this group of patients (RME = 0.01, *P* value < 0.001 for all comparisons). However, its expression was higher in male patients with aura compared with those without aura (*P* value < 0.001).

Expression of *SOCS3* was lower in patients without aura compared with controls (RME = 0.07, *P* value < 0.001), and in both sex-based subgroups (RME = 0.09, *P* value = 0.011 and RME = 0.07, *P* value = 0.001 for females and males, respectively). On the other hand, *SOCS3* was up-regulated in patients with aura compared with those without aura (RME = 7.46, *P* value = 0.001), and in both female and male patients with aura compared with sex-matched patients without aura (RME = 7.68, *P* value = 0.011, and RME = 6.92, *P* value = 0.001 for females and males, respectively).

*SOCS5* was down-regulated in patients without aura compared with controls (RME = 0.10, *P* value < 0.001). This pattern was also detected among female and male subgroups (*P* value = 0.003 and 0.001 for females and males, respectively). Expression of this gene was also lower in patients with aura compared with controls (RME = 0.03, *P* value <0.001), and in male patients of this group compared with controls (RME = 0.03, *P* value = 0.004). However, *SOCS5* was up-regulated in male patients with aura compared with sex-matched patients without aura (RME = 6.67, *P* value = 0.001). Table 2 shows the results of *post hoc* analysis (Dunn’s test).

**Table 2:** The results of *post hoc* analysis (Dunn’s test).

	Total			Females			Males		
Gene	Adjusted *P* Value (Control vs. Aura)	Adjusted *P* Value (Control vs. Without Aura)	Adjusted *P* Value (Aura vs. Without Aura)	Adjusted *P* Value (Control vs. Aura)	Adjusted *P* Value (Control vs. Without Aura)	Adjusted *P* Value (Aura vs. Without Aura)	Adjusted *P* Value (Control vs. Aura)	Adjusted *P* Value (Control vs. Without Aura)	Adjusted *P* Value (Aura vs. Without Aura)
*SOCS1*	0.032	0.000	0.037	0.070	0.010	0.105	0.277	0.113	0.113
*SOCS2*	0.000	0.000	0.097	0.000	0.000	0.138	0.002	0.000	0.000
*SOCS3*	0.049	0.000	0.001	0.198	0.012	0.011	0.379	0.001	0.001
*SOCS5*	0.000	0.000	0.180	0.004	0.003	0.551	0.347	0.001	0.001

Expressions of both *SOCS1* and *SOCS5* were higher in male migraineurs with aura compared with female migraineurs with aura. No other significant difference was detected in the expression of these genes between males and females.

**Figure 2:**
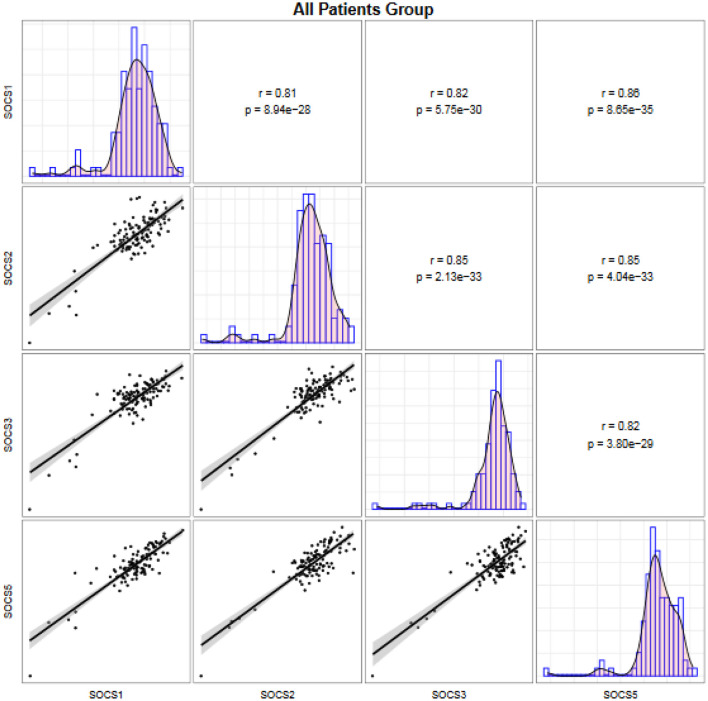
Correlation between transcript levels of the *SOCS* genes among patients. Distributions of expression data of the *SOCS* gene are presented on the diagonal. The bivariate scatter plots are shown on the bottom of the diagonal. The upper part of the diagonal shows correlation coefficients (r) and *P* values.

**Figure 3:**
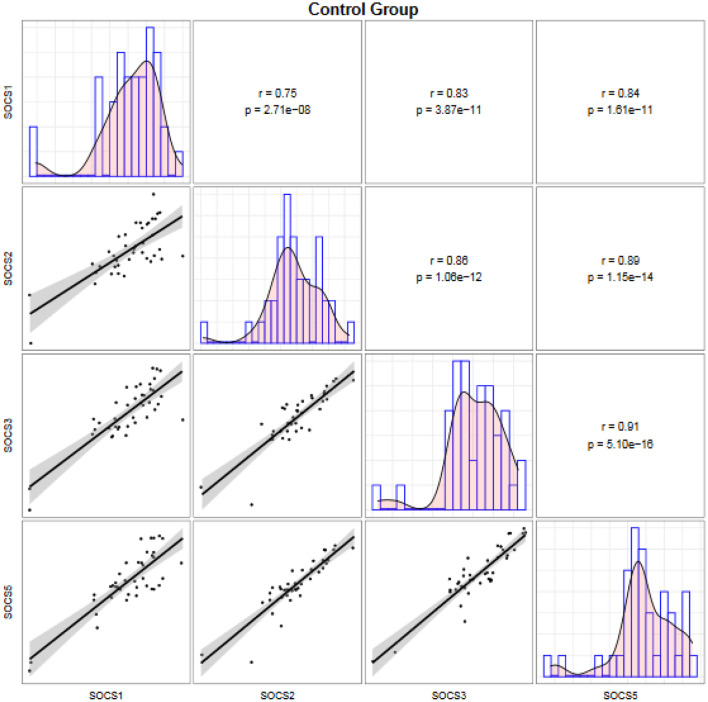
Correlation between transcript levels of the *SOCS* genes among controls. Distributions of expression data of the *SOCS* gene are presented on the diagonal. The bivariate scatter plots are shown on the bottom of the diagonal. The upper part of the diagonal shows correlation coefficients (r) and *P* values.

Expressions of *SOCS* genes were significantly correlated with each other both among migraineurs (Figure 2) and among controls (Figure 3).

**Figure 4:**
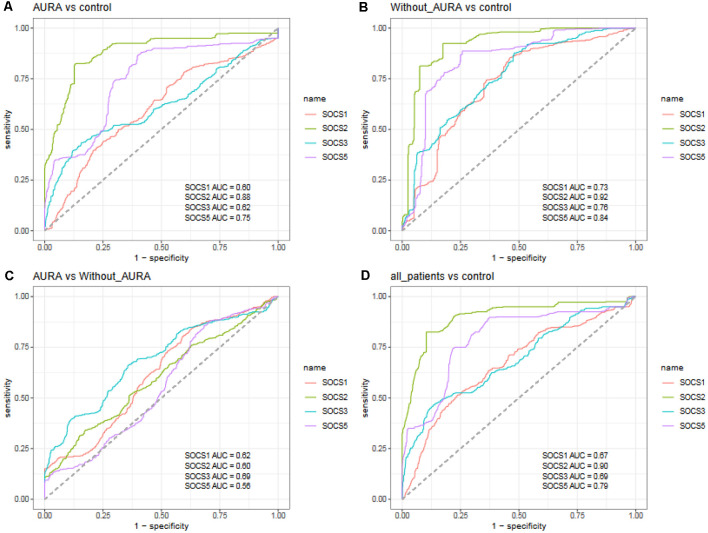
Receiver operating characteristic (ROC) curves for assessment of the appropriateness of *SOCS* genes for separation of migraineurs with aura from controls **(A)**, migraineurs without aura from controls **(B)**, patients with aura from those without aura **(C)**, and total migraineurs from controls **(D)**.

In order to assess the ability of the *SOCS* genes in the separation of migraineurs from controls as well as the separation of two groups of migraineurs, we depicted ROC curves (Figure 4).

*SOCS2* could differentiate patients with aura from controls with AUC, sensitivity, and specificity values of 0.88, 0.82, and 0.87, respectively. This gene could also separate patients without aura from controls with AUC, sensitivity and specificity values of 0.92, 0.92, and 0.83, respectively. When assessing its ability to separate total patients from total controls, these values were 0.90, 0.83, and 0.90, respectively. *SOCS5* could separate patients without aura from controls with AUC, sensitivity, and specificity values of 0.84, 0.89, and 0.74, respectively. The combination of transcript amounts of four genes has enhanced AUC values to 0.91, 0.93, 0.66, and 0.92 for separation of patients with aura from controls, patients without aura from controls, patients with aura from those without aura, and total patients from controls, respectively (Table 3).

**Table 3:** Area under curve (AUC), sensitivity, and specificity values of *SOCS* transcripts in separation between two classes of migraineurs as between total cases and controls.

Number of Samples	*SOCS1*			*SOCS2*			*SOCS3*			*SOCS5*			All genes		
	AUC	Sensitivity	Specificity	AUC	Sensitivity	Specificity	AUC	Sensitivity	Specificity	AUC	Sensitivity	Specificity	AUC	Sensitivity	Specificity
Aura/Controls
Total 63/40	0.60	0.44	0.75	0.88	0.82	0.87	0.62	0.44	0.84	0.75	0.87	0.60	0.91	0.89	0.84
Without aura/Controls
Total 53/40	0.73	0.85	0.54	0.92	0.92	0.83	0.76	0.88	0.52	0.84	0.89	0.74	0.93	0.83	0.94
Aura/Without aura
Total 63/53	0.62	0.84	0.37	0.60	0.34	0.83	0.69	0.66	0.64	0.56	0.88	0.30	0.66	0.49	0.80
All Patients/Controls
Total 116/40	0.67	0.51	0.76	0.90	0.83	0.90	0.69	0.47	0.87	0.79	0.75	0.78	0.92	0.95	0.83

## Discussion

Migraine is a complex disorder with possible immunologic background (Bruno et al., 2007). We have recently reported over-expression of INF-γ, IL-4, TGF-β, and TNF-α, while down-regulation of CXCL8 in migraineurs compared with controls (Taheri et al., 2021). Moreover, we have demonstrated down-regulation of two members of protein inhibitor of activated STAT (PIAS) family in these patients (Ghafouri-Fard et al., 2021), further suggesting the abnormal immune homeostasis among migraineurs. In the current study, we showed down-regulation of *SOCS1–3* and *SOCS5* in migraineurs compared with healthy controls. SOCS1 has been shown to be an important inhibitor of INF-γ signaling (Alexander et al., 1999), a cytokine which is up-regulated in migraineurs (Taheri et al., 2021). Down-regulation of SOCS1/3 has been shown to promote the expression of IFN-α/β in oligodendroglial cells (Li et al., 2021). Meanwhile, IFN-β has been shown to aggravate or induce headaches in certain settings (Elmazny et al., 2020). SOCS2 has been found to regulate neurotrophin receptor (Trk) signaling (Uren and Turnley, 2014), a pathway which is implicated in induction and modulation of nociceptive routes (Martins et al., 2017). Moreover, SOCS3 has a role in ubiquitination and degradation of IDO (Orabona et al., 2005, 2008), a protein with substantial contribution in the induction of neuropathic pain (Rojewska et al., 2018). Thus, down-regulation of *SOCS* genes can contribute to the pathoetiology of migraines from different routes.

Yet, sex-based subgroup analyses indicated non-significant results in some comparisons. Although the main cause of this observation is not clear, the impact of estradiol on the induction of SOCS2 signaling (Santana-Farre et al., 2008) might explain this finding.

Notably, expressions of *SOCS1* and *SOCS3* transcripts were higher in patients with aura compared with those without aura. Migraine aura has been shown to have resulted from disturbances of the cerebral cortex, alterations in brain blood, sustained nerve cell depression, disturbance in brain ion homeostasis, and increased energy metabolism (Lauritzen, 1994). The underlying cause of differential expression of *SOCS* transcripts between migraineurs with and without aura should be assessed in future investigations.

Among *SOCS* transcripts, the best AUC values have been detected for *SOCS2*, suggesting its superior function as a peripheral marker for migraine. A combination of transcript levels of *SOCS* genes could appropriately separate migraineurs from healthy controls. Yet, this approach was not suitable for the separation of migraineurs with aura from those without aura.

Taken together, the present study suggested a global dysregulation of *SOCS* transcripts among migraineurs and provided further evidence of the contribution of immune responses in the pathoetiology of this neurologic condition. It is worth mentioning that attention to migraine subtype and some migraine features, such as frequency, is important because the expression level of the involved genes may be different in patients with chronic and episodic migraines, as it was different among patients with and without aura. So, we recommend further assessment of expression of these genes in association with other characteristics of migraine attacks.

## Data Availability Statement

The raw data supporting the conclusions of this article will be made available by the authors, without undue reservation.

## Ethics Statement

The studies involving human participants were reviewed and approved and the study protocol was verified by the ethical committee of Shahid Beheshti University of Medical Sciences. The patients/participants provided their written informed consent to participate in this study.

## Author Contributions

MT and SG-F wrote the draft and revised it. KH and MS performed the experiment and clinical assessment. AS analyzed the data. All authors contributed to the article and approved the submitted version.

## Conflict of Interest

The authors declare that the research was conducted in the absence of any commercial or financial relationships that could be construed as a potential conflict of interest.

## Publisher’s Note

All claims expressed in this article are solely those of the authors and do not necessarily represent those of their affiliated organizations, or those of the publisher, the editors and the reviewers. Any product that may be evaluated in this article, or claim that may be made by its manufacturer, is not guaranteed or endorsed by the publisher.
